# Comparing the Neuroprotective Effects of Telmisartan, Perindopril, and Nebivolol Against Lipopolysaccharide-Induced Injury in Neuron-Like Cells

**DOI:** 10.7759/cureus.27429

**Published:** 2022-07-29

**Authors:** Selçuk Şen, Ebru Hacıosmanoğlu

**Affiliations:** 1 Department of Medical Pharmacology, Istanbul Faculty of Medicine, Istanbul University, Istanbul, TUR; 2 Department of Biophysics, Faculty of Medicine, Bezmialem Vakif University, Istanbul, TUR

**Keywords:** neurodegeneration, acetylcholine, antihypertensive, neuroprotective, nebivolol, perindopril, telmisartan

## Abstract

The effect of antihypertensive drugs, especially drugs modulating the renin-angiotensin-aldosterone-system (RAAS), on neurodegenerative diseases still needs to be investigated. This study aimed to compare the effects of three different antihypertensive drugs (telmisartan, perindopril, and nebivolol) on neuroprotection and acetylcholine (ACh) levels against lipopolysaccharide (LPS)-induced injury in a differentiated SH-SY5Y cell line. Cells were treated with retinoic acid for differentiation to a neuronal phenotype. LPS 20 (μg/mL) was applied to the cells for one hour. Then, the cells were treated with 1, 5, and 10 µg/mL concentrations of telmisartan, perindopril, and nebivolol separately for 24 hours, except for the control and LPS alone groups. Cell viability was evaluated using a 3-(4,5-dimethylthiazol-2-yl)-2,5-diphenyltetrazolium bromide (MTT) assay. ACh levels were analyzed using an enzyme immunosorbent assay in the culture medium. Tumor necrosis factor-alpha (TNF-α), interleukin 1 beta (IL-1β), and nuclear factor kappa-light-chain-enhancer of activated B cells (NFκB) expressions were evaluated using western blot analysis. Telmisartan demonstrated the highest cell viability against LPS-induced injury, whereas the protective effect of perindopril was moderate. Nebivolol showed no neuroprotective effect. The protective effect of 10-µg/mL telmisartan was superior to 10 µg/mL perindopril (p=0.006), 5 µg/mL perindopril (p=0.001), 1 µg/mL perindopril (p=0.001), and 1, 5, and 10 µg/mL nebivolol (p<0.001). Among all the study drugs, only telmisartan provided a statistically significant increase in ACh levels after LPS-induced injury. Additionally, the administration of telmisartan provided a concentration-dependent reduction in TNF-α, IL-1β, and NFκB expression against LPS-induced neuroinflammation. These findings suggest that telmisartan has a superior neuroprotective effect against LPS-induced injury in neuron-like cells compared with both perindopril and nebivolol.

## Introduction

In addition to the association with increasing age, it has been shown that cardiovascular diseases and hypertension are important and independent risk factors for cognitive impairment and dementia [1,2]. The renin-angiotensin-aldosterone system (RAAS) is a complex system with many metabolites and plays an essential role in blood pressure regulation, electrolyte balance, and the development of hypertension. However, the effects of RAAS are not limited to the regulation of blood pressure and electrolyte balance. Systemic and local effects of RAAS contribute to many neuronal, hormonal, and metabolic effects. In this sense, several clinical and pre-clinical studies have investigated the role of RAAS and its inhibition of cognitive impairment and dementia [3]. Angiotensin-converting enzyme (ACE) inhibitors and angiotensin receptor blockers (ARBs) can be counted as classic RAAS inhibitors. It was demonstrated that lowering blood pressure with RAAS inhibitors reduced the risk of cognitive impairment and dementia [3]. However, the underlying mechanism is currently not well-known. RAAS-blocking antihypertensive drugs inhibit the system at different points and thus differ from each other in their mechanism of action [4]. ARBs block angiotensin II AT-1 receptors and also allow angiotensin II to bind to AT-2 receptors, which have neuroprotective properties [5]. Regarding their mechanism of action, ARBs may have superior neuroprotective effects than other antihypertensive drugs, including ACE inhibitors. In contrast to RAAS inhibitors, the effect of beta-blockers, especially the selective and central nervous system (CNS) active β1 receptor blockers, on cognitive functions is controversial, and some clinical studies have shown that administration of selective beta-blockers can worsen cognitive functions [6,7].

Acetylcholine (ACh) has an essential role in the pathophysiology of dementia and Alzheimer’s disease (AD). The results of post-mortem studies have shown that lower levels of ACh are associated with AD [8]. Several inhibitors of ACh esterase, which is an enzyme responsible for ACh breakdown, are approved for the treatment of AD. There is a hypothesis that is mainly based on a possible increase in ACh release by the effect of ARBs via AT1 receptor blockade and stimulation of AT2 [5].

In our study, we selected an ARB, an ACE inhibitor, and a beta-blocker agent that can cross the blood-brain barrier (BBB) to compare their neuroprotective effects. In order to provide useful information for future neuroscience research, we chose telmisartan, perindopril, and nebivolol because all these drugs have lipophilic properties and can cross the BBB. Additionally, all these drugs are very commonly used in daily clinical practice. Telmisartan is an angiotensin II AT1 receptor blocker, whereas perindopril is an ACE inhibitor that inhibits the conversion of angiotensin I to angiotensin II. The last drug in the present in vitro experiment was nebivolol, which is a beta-blocker that selectively blocks the β1 receptor in the sympathetic nervous system and inhibits renin release in the juxtaglomerular apparatus.

In the present study, we aimed to compare the effects on ACh release and the neuroprotective effects (based on cell viability) of three different classes of antihypertensive drugs against lipopolysaccharide (LPS)-induced injury in a differentiated human SH-SY5Y cell line (differentiated to a neuronal phenotype), which provided an appropriate model to evaluate the neuroprotective effects of different compounds [9,10]. Additional experiments for cell viability were repeated in the absence of LPS stimulation. To the best of our knowledge, the present study is the first to compare the neuroprotective effects of three different classes of antihypertensives in the same in vitro settings.

## Materials and methods

Cell culture, differentiation, and treatment

The human neuroblastoma SH-SY5Y cell line was obtained from the American Type Culture Collection (ATCC; cat. no. CRL-2266; Manassas, Virginia, USA). Cells were cultured under humidified 5% CO_2_ and 95% air in DMEM/F12 medium with L-glutamine (Gibco; Thermo Fisher Scientific, Inc.) containing 10% fetal bovine serum (FBS, Gibco; Thermo Fisher Scientific, Inc.), 1% penicillin-streptomycin (Gibco; Thermo Fisher Scientific, Inc.), and 1% amphotericin B (Gibco; Thermo Fisher Scientific, Inc.) at 37 °C. The medium was changed twice per week, and cells were split at about 80% confluence. Cells were seeded in 96-well plates (3 × 10^3^ cells/per well), 24-well plates (3 × 10^5^ cells/per well), and 6-well plates (6 × 10^5^ cells/per well) for the experiments [11]. Cells to be treated with retinoic acid (RA, Merck, St. Louis, Missouri, USA) were for differentiation to a neuronal phenotype in serum-free neurobasal medium (Thermo Fisher, Waltham, Massachusetts, USA) with 1% penicillin-streptomycin for one week in the dark. The culture medium was changed every three to four days, supplemented with fresh additives of RA [12]. Before the treatment, 20 μg/mL LPS (Merck, St. Louis, Missouri, USA) was applied to cells for one hour. Then, the cells were treated with different concentrations of telmisartan, perindopril, and nebivolol (1, 5, and 10 µg/mL) separately for 24 hours, except for the LPS alone group. All drugs (telmisartan, perindopril, and nebivolol, provided by Neutec R&D) were dissolved in dimethyl sulfoxide (DMSO; Merck, St. Louis, Missouri, USA). Additional experiments for cell viability were repeated in the absence of LPS stimulation.

Study groups

For the primary aim, the study was divided into ten groups: the LPS alone group, three nebivolol treatment groups, three perindopril treatment groups, and three telmisartan treatment groups. In the treatment groups, in addition to LPS (20 µg/mL), nebivolol, perindopril, and telmisartan were administrated in concentrations of 1, 5, and 10 µg/mL separately. Additional experiments for cell viability were repeated in the absence of LPS stimulation and were grouped as a control, three nebivolol treatment groups (1, 5, and 10 µg/mL), three perindopril treatment groups (1, 5, and 10 µg/mL), and three telmisartan treatment groups (1, 5, and 10 µg/mL).

In addition to direct comparison with control and LPS groups separately for each study drug, the cell viability (%) results of the study drugs against LPS-induced injury were compared with each other using statistical methods for multiple comparisons described in the statistical analysis section.

3-(4,5-Dimethylthiazol-2-yl)-2,5-diphenyltetrazolium bromide (MTT) cell viability assay

The viability of cells in each group with or without administration of LPS and/or study drugs was tested using an MTT assay. Cells were incubated at 37 °C with 0.1 mg/mL MTT (Merck, St. Louis, Missouri, USA) for two hours. The supernatants were decanted without being dispensed to the cells. Then, 100 µL DMSO (Merck) was added to the cells, which were then kept in the dark. After 30 minutes, optical densities were measured using a microplate reader (BioTek, Winooski, Vermont, USA) at 570 nm [13]. The cell viability assay for each group was performed in triplicate.

ACh ELISA assay

An ACh enzyme-linked immunosorbent assay (ELISA) was performed according to the manufacturer’s instructions to detect the ACh release in the culture medium. Briefly, 3 × 10^5^ cells in each group were cultured using a differentiation medium (choline-free medium). The cells were treated with 20 µg/mL LPS (except the control group) for one hour, and study drugs (except the control and LPS alone groups) were added separately for 24 hours. The relative concentration of ACh in the culture medium was calculated using an ACh ELISA Kit (Abcam, Cambridge, UK) with a microplate reader (BioTek, Winooski, Vermont, USA) at 570 nm. Two parallel wells were repeated in each group.

SDS-PAGE and western blotting

Inflammation-related markers were evaluated using Western blot analysis after sodium dodecyl sulfate-polyacrylamide gel electrophoresis (SDS-PAGE) [14]. After SHSY-5Y cells (10^6^/well) were differentiated into neurons with RA, they were treated with telmisartan, perindopril, and nebivolol for 24 hours. Total protein was isolated using the RIPA Lysis Buffer (Santa Cruz Biotechnology, Dallas, Texas, USA) and then protein concentrations were determined using a Bradford assay (Merck). Samples were subjected to western blot analysis as previously described [15]. Equal amounts (40 µg) of protein were resolved on SDS-PAGE using a 12% separation gel, and the gels were transferred to a polyvinyl difluoride membrane (Bio-Rad, CA, USA). The membrane was blocked with 5% non-fat milk in a Tris-buffered saline Tween (TBST) buffer for one hour at room temperature. Membranes were incubated with primary antibody interleukin (IL)-1β (rabbit mAb; 1:1000; cat. no: 12703; Cell Signaling Technology, Inc.), nuclear factor kappa-light-chain-enhancer of activated B (NFκB) (rabbit mAb; 1:1000; cat. no: 8242; Cell Signaling Technology, Inc.), tumor necrosis factor-alpha (TNF-α) (rabbit mAb; 1:1000; cat. no: 6945; Cell Signaling Technology, Inc.), and β-actin (mouse mAb; 1:1000; cat. no: 3700; Cell Signaling Technology, Inc.), overnight at 4 °C separately. β-actin protein levels served as a loading control. After incubation with horseradish peroxidase (HRP)-conjugated goat anti-mouse or goat anti-rabbit secondary antibody (1:5000; cat. nos. 7076 or 7074; Cell Signaling Technology, Inc.) for one hour, blots were developed using enhanced chemiluminescence (ECL, Elabscience, Houston, Texas, USA) with the Fusion FX7 system (Vilber Lourmat, France). The quantification of protein bands was analyzed using ImageJ software (version 1.4.3.67; National Institutes of Health).

Statistical analysis

Statistical analyses were conducted using the IBM Statistical Package for the Social Sciences (SPSS) for Windows version 21.0 software. Multiple comparisons between the treatment groups were performed using a one-way analysis of variance (ANOVA) followed by Tukey’s post-hoc test. For correlation analysis, Pearson's correlation coefficient was used. P-values of <0.05 were considered significant. Data are presented as mean ± standard deviation (SD), and the results of Western blot analysis are given as mean ± standard error of the mean (SEM).

## Results

The effect of telmisartan, perindopril, and nebivolol on cell viability in the differentiated SH-SY5Y cell line

**Figure 1:**
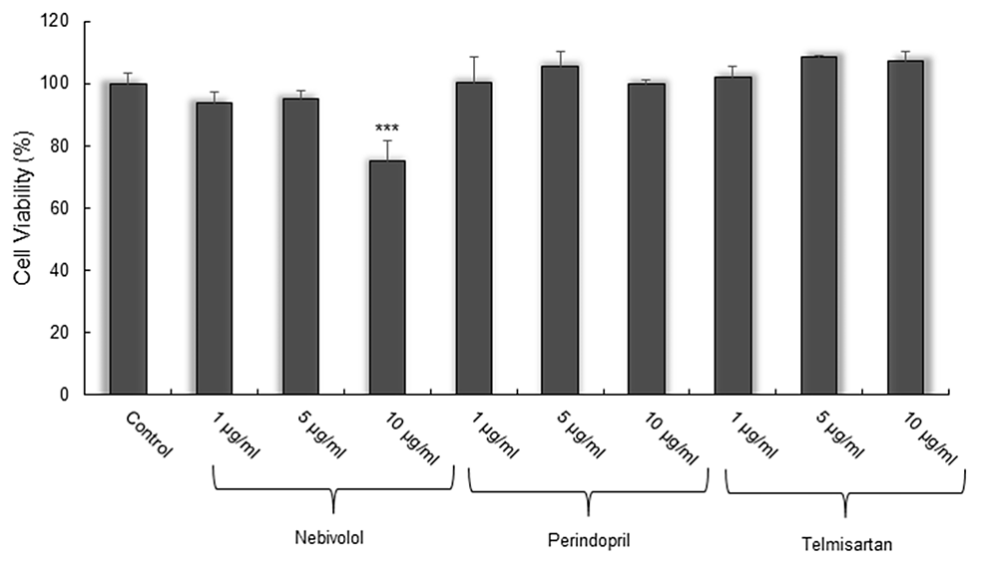
Effects of nebivolol, perindopril, and telmisartan on cell viability Cells were exposed to 1, 5, or 10 μg/mL of nebivolol, perindopril, and telmisartan for 24 hours. Cell viability was expressed as a percentage. The control (0 μM) was set as 100%. Each sample was measured in triplicate. Values are means ± SD of three independent experiments. ***p<0.001 compared with the control.

In the first step, the effects of study drugs on cell viability were evaluated in the differentiated SH-SY5Y cell line, without LPS stimulation. The percentages of cell viability for 1, 5, and 10 µg/mL nebivolol were 93.86±3.28%, 95.19±4.00%, and 74.99±3.19%, respectively. The percentages of cell viability for 1, 5, and 10-µg/mL perindopril were 100.36±2.53%, 105.73±1.71%, and 99.85±6.24%, respectively. The cell viability percentages for 1, 5, and 10 µg/mL telmisartan were 102.25 ± 11.52%, 108.49±2.58%, and 107.37±1.57%, respectively. Among all the study drugs, only 10 µg/mL of nebivolol caused a significant decrease in cell viability compared with the control (p<0.001) (Figure 1).

The effect of telmisartan on cell viability against LPS-induced injury in the differentiated SH-SY5Y cell line

**Figure 2:**
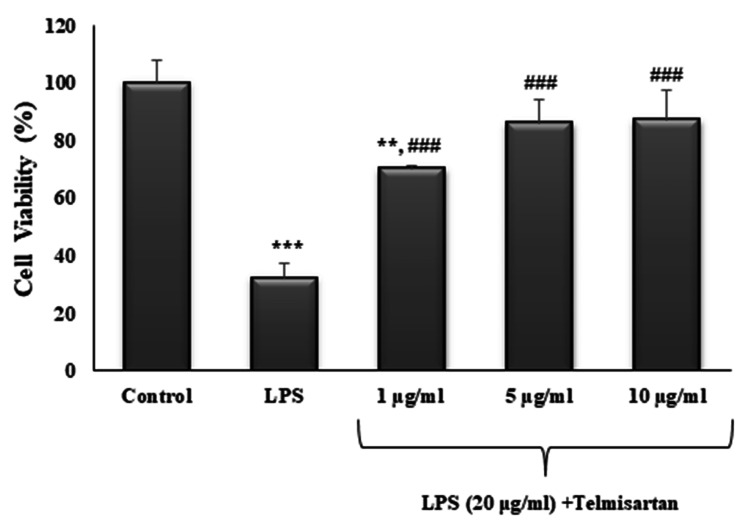
The effect of telmisartan on cell viability against LPS-induced injury Cells were pre-treated with LPS (20 μg/mL) for one hour and exposed to 1, 5, or 10 μg/mL of telmisartan for 24 hours. Cell viability was expressed as a percentage. The control (0 μM) was set as 100%. Each sample was measured in triplicate. Values are means ± SD of three independent experiments. **p<0.01, ***p<0.001 compared with the control; ^###^p<0.001 compared with LPS.

As expected, the administration of LPS alone significantly decreased the cell viability in comparison with the control (p<0.001). The potential neuroprotective effect of telmisartan on LPS-induced cell death in the differentiated SH-SY5Y cell line was investigated. When compared with the administration of LPS alone, the addition of telmisartan in increasing concentrations (1, 5, and 10 µg/mL) significantly reversed the decrease in cell viability caused by LPS (for all concentrations, p<0.001). Additionally, telmisartan in 5 and 10-µg/mL concentrations provided cell viability values similar to the control. The percentages of cell viability for LPS alone and LPS plus 1, 5, and 10 µg/mL telmisartan were 32.24±5.18%, 70.10±1.32%, 86.01±8.22%, and 87.11±10.49%, respectively (Figure 2).

The effect of perindopril on cell viability against LPS-induced injury in the differentiated SH-SY5Y cell line

**Figure 3:**
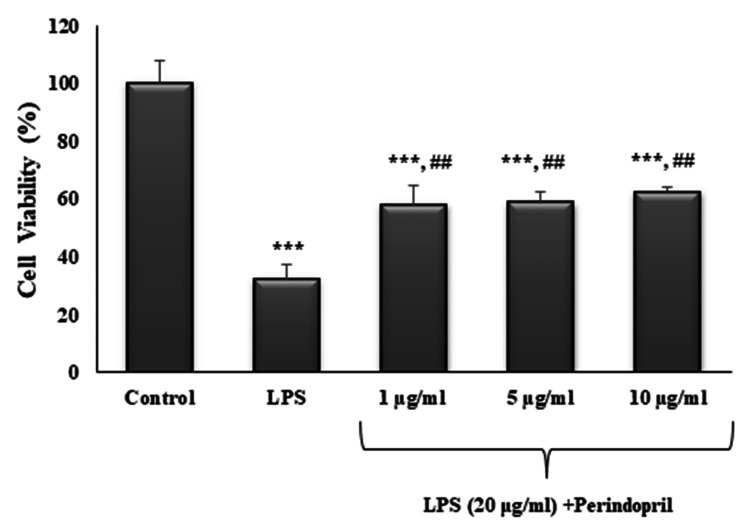
The effect of perindopril on cell viability against LPS-induced injury Cells were pre-treated with LPS (20 μg/ml) for one hour and exposed to 1, 5, or 10 μg/ml of perindopril for 24 hours. Cell viability was expressed as a percentage. The control (0 μM) was set as 100%. Each sample was measured in triplicate. Values are means ± SD of three independent experiments. ***p<0.001 compared with the control; ^##^p<0.01 compared with LPS.

The potential effect of perindopril on LPS-induced cell death in the differentiated SH-SY5Y cell line was evaluated. The percentages of cell viability for LPS alone and LPS plus 1, 5, and 10 µg/mL perindopril were 32.24±5.18%, 57.89±6.67%, 58.85±3.70%, and 62.41±1.90%, respectively (Figure 3). The addition of perindopril to the LPS-induced cells significantly increased the viability at all doses (p=0.004 for 1 µg/mL perindopril, p=0.003 for 5 µg/mL perindopril, p=0.001 for 10 µg/mL perindopril). However, there were no significant differences between the concentrations.

The effect of nebivolol on cell viability against LPS-induced injury in the differentiated SH-SY5Y cell line

**Figure 4:**
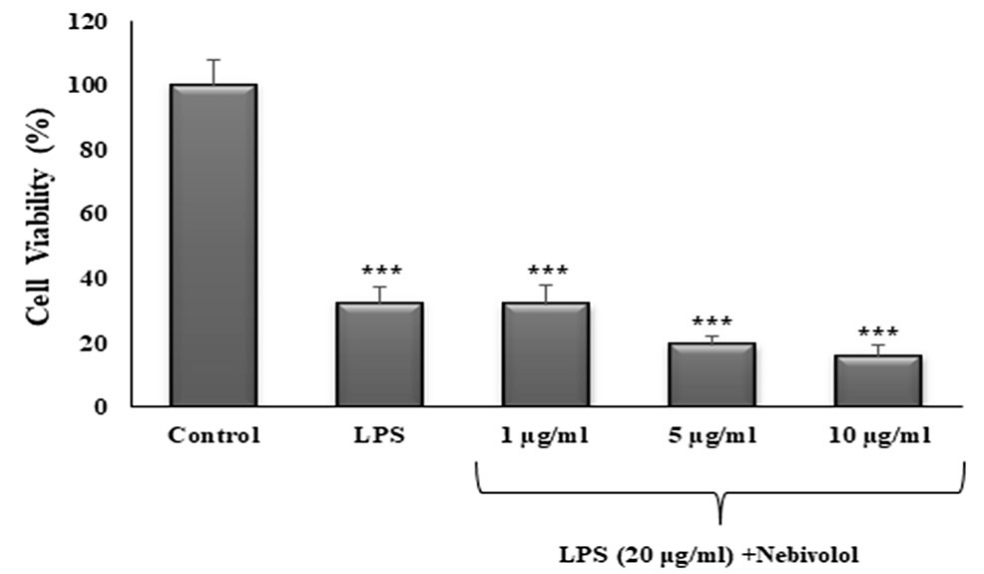
The effect of nebivolol on cell viability against LPS-induced injury Cells were pre-treated with LPS (20 μg/mL) for one hour and exposed to 1, 5, or 10 μg/mL of nebivolol for 24 hours. Cell viability was expressed as a percentage. The control (0 μM) was set as 100%. Each sample was measured in triplicate. Values are means ± SD of three independent experiments. ***p<0.001 compared with the control.

The effect of nebivolol in the differentiated SH-SY5Y cell line was in contrast to the neuroprotective effects of telmisartan and perindopril. The addition of nebivolol to the LPS-induced cell line did not increase the viability. Additionally, the 5 and 10 µg/mL concentrations of nebivolol decreased the viability when compared with LPS alone, even though it was statistically nonsignificant (p>0.05). The percentages of cell viability for LPS alone and LPS plus nebivolol 1, 5, and 10 µg/mL were 32.24±5.18%, 31.96±5.97%, and 19.34±2.57%, 15.91±3.55%, respectively (Figure 4).

Overall comparison of the neuroprotective effects of telmisartan, perindopril, and nebivolol against LPS-induced injury

All the study drugs differed from each other in their neuroprotective effects against LPS-induced injury. Therefore, the overall difference was compared. Telmisartan in all concentrations demonstrated the highest cell viability, whereas the lowest cell viability was detected with nebivolol. The cell viability (%) value of 10 µg/mL telmisartan was significantly higher than the values of 10 µg/mL perindopril (p=0.006), 5 µg/mL perindopril (p=0.001), 1 µg/mL perindopril (p=0.001), and 1 µg/mL, 5 µg/mL, and 10 µg/mL nebivolol (p<0.001). There was no statistically significant difference between the different concentrations of telmisartan; however, the cell viabilities with both telmisartan 5 and 10 µg/mL were slightly higher than with telmisartan 1 µg/mL.

The effect of telmisartan, perindopril, and nebivolol on ACh levels after LPS-induced injury in the differentiated SH-SY5Y cell line

**Figure 5:**
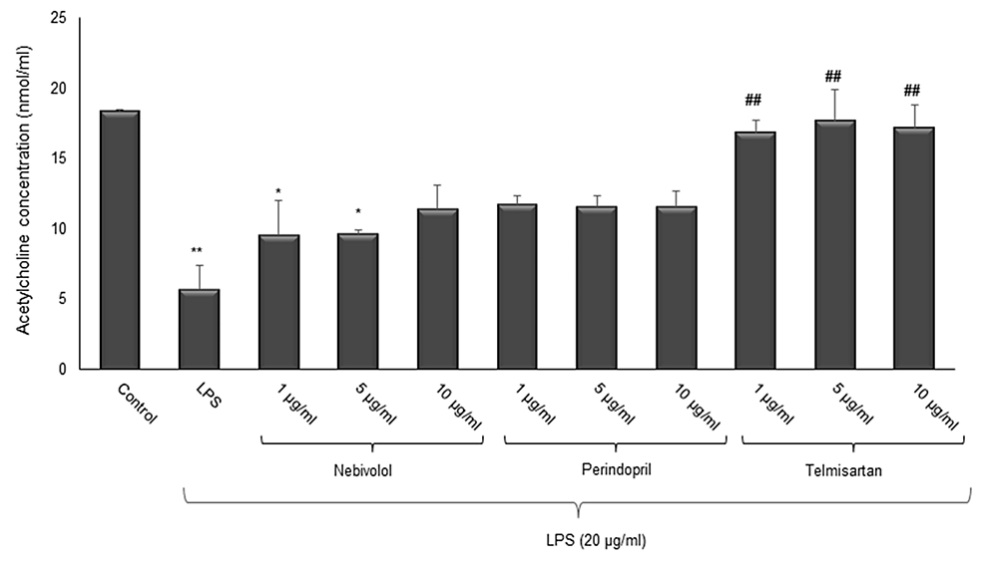
The effects of nebivolol, perindopril, and telmisartan on ACh levels Cells were pre-treated with LPS (20 μg/mL) for one hour, except the control group, and exposed to 1, 5, or 10 μg/mL of nebivolol, perindopril, and telmisartan in each treatment group separately for 24 hours, except the control and LPS alone groups. Values are presented as means ± SD of independent experiments. *p<0.05, **p<0.01 compared with the control; ^##^p<0.01 compared with LPS alone.

In addition to cell viability, the effect of study drugs on ACh levels was evaluated under the condition of LPS-induced injury in the differentiated SH-SY5Y cell line. Administration of LPS alone considerably lowered the ACh levels (p=0.001). After LPS-induced injury, only the administration of telmisartan provided a statistically significant increase in ACh levels; the administration of perindopril or nebivolol in different concentrations showed no statistically significant changes (Figure 5). The ACh levels in the control and LPS alone groups were 18.42±0.015 nmol/mL and 5.69±2.674 nmol/mL, respectively. The ACh levels in the 1, 5, and 10 µg/mL telmisartan groups were 16.86±0.852 nmol/mL, 17.74±3.153 nmol/mL, and 17.24±1.602 nmol/mL, respectively. The ACh levels in the 1, 5, and 10 µg/mL perindopril groups were 11.74±0.567 nmol/mL, 11.58±0.785 nmol/mL, and 11.59±1.092 nmol/mL, respectively. Lastly, the ACh levels in the 1, 5, and 10 µg/mL nebivolol groups were 9.54±2.467 nmol/mL, 9.66±0.269 nmol/mL, and 11.40±2.670 nmol/mL, respectively. In addition to its direct protective effects on neurons, telmisartan also provided maintenance of ACh levels differently from perindopril and nebivolol.

**Figure 6:**
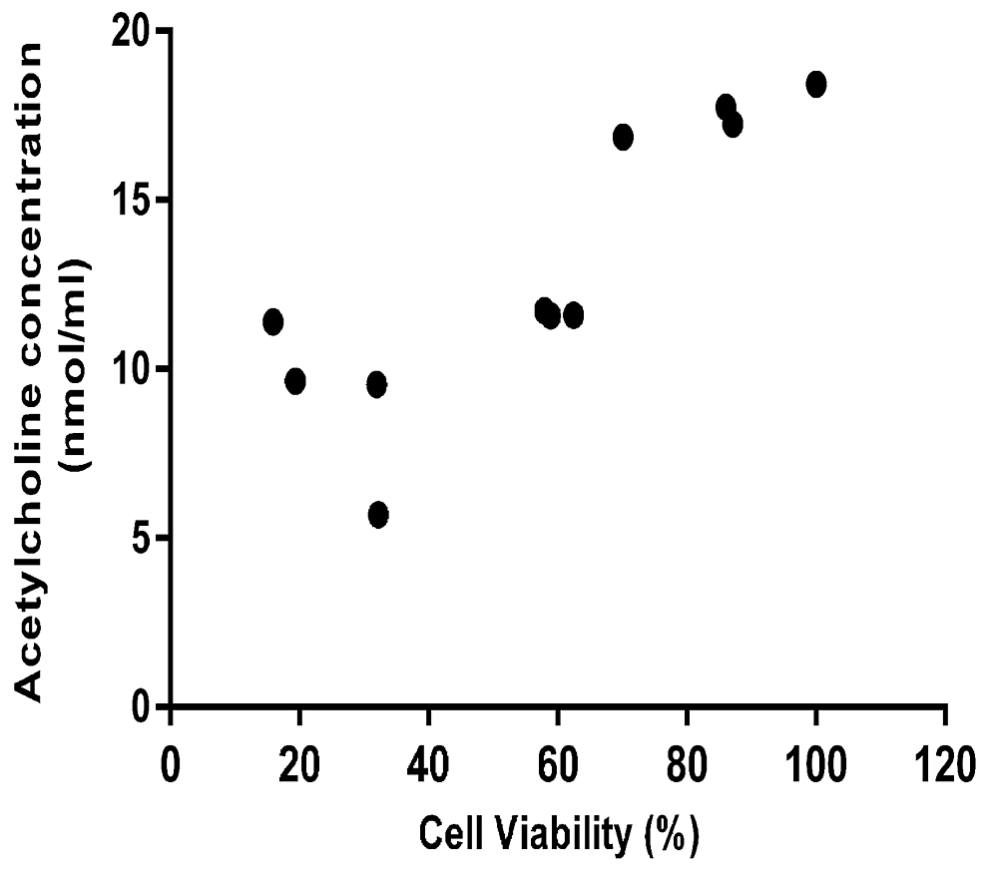
The correlation between ACh concentrations and cell viability The mean values of cell viability and ACh levels were calculated for each group separately. A strong linear relationship between the mean cell viability and mean ACh level was detected using Pearson’s correlation analysis (r=0.848, p=0.001).

The correlation between the mean ACh level and the mean cell viability was also evaluated. As expected, there was a strong linear relationship between these two parameters (r=0.848, p=0.001) (Figure 6).

Decrease in the inflammation-related protein expressions with telmisartan after LPS-induced neuroinflammation in the differentiated SH-SY5Y cell Line

**Figure 7:**
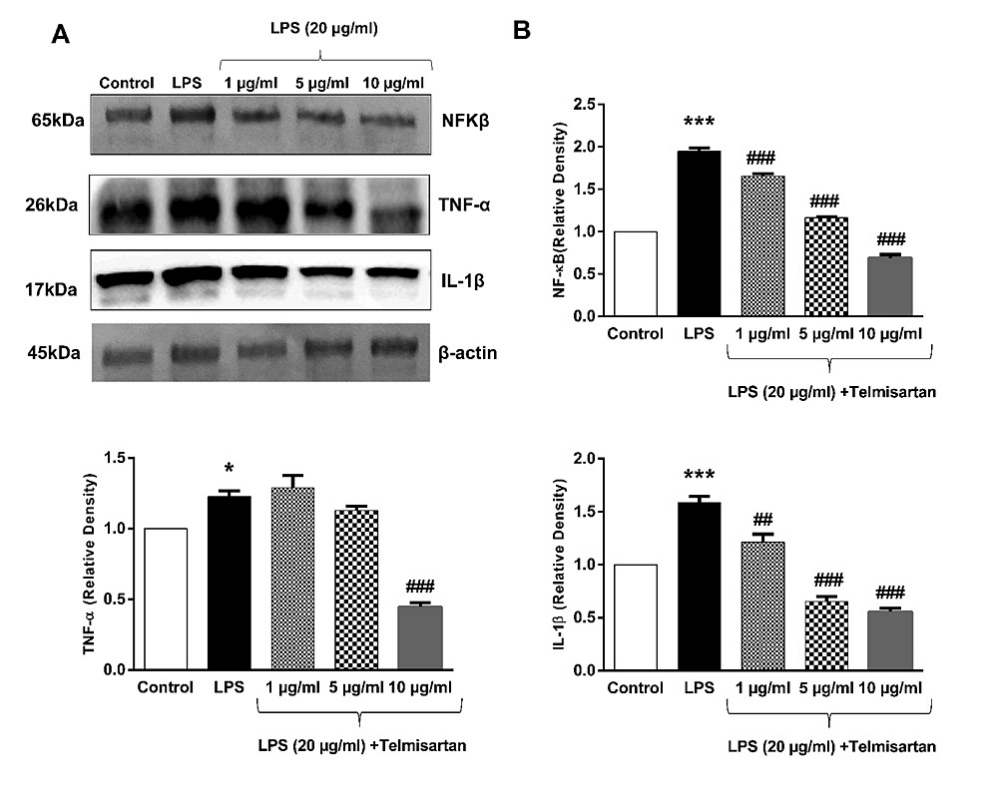
Effects of telmisartan on TNF-α, IL-1β, and NFKβ expression in LPS-induced neuroinflammation (A) Proteins extracted from differentiated SH-SY5Y cells pretreated with 20 μg/mL LPS for one hour and exposed to 1, 5, 10 µmol/mL telmisartan for 24 hours were subjected to Western blot analysis. (B) Protein expression levels of TNF-α, IL-1β, and NFKβ were analyzed using the ImageJ. Values are expressed as means ± SEM of three experiments. *p<0.05, ***p<0.001 compared with the control; ^##^p<0.01, ^###^p<0.001 compared with LPS.

Inflammation-related protein expressions were evaluated using Western blot analysis after SDS-PAGE. The expressions of TNF-α, IL-1β, and NFκB were evaluated to understand the underlying mechanisms related to inflammatory processes. Cells were treated with LPS for one hour in the presence or absence of telmisartan. LPS stimulation significantly elevated the levels of these proteins (Figure 7). Treatment with telmisartan led to a dose-dependent inhibition of LPS-induced production of TNF-α, IL-1β, and NFκB. Telmisartan at 10 μg/mL led to a reduction in TNF-α, IL-1β, and NFκB expressions by 58.3%, 63%, and 59.4%, respectively, compared with LPS alone (Figure 7). No significant changes in TNF-α, IL-1β, and NFκB expressions were detected with the administration of nebivolol or perindopril in different concentrations.

## Discussion

Hypertension is an independent risk factor for cerebrovascular diseases and neurodegeneration [16,17]. Its key role in the development of hypertension and many systemic and local effects makes the research on RAAS an important scientific field. Therefore, investigating the effect of RAAS-blocking antihypertensive drugs on the CNS and neurodegeneration has become a very promising research topic. The findings in this field have demonstrated that lowering blood pressure with RAAS-blocking agents can be a neuroprotective strategy. The protective effects of RAAS inhibitors against cognitive impairment have been reported in observational clinical studies [18,19]. However, there is still a need to understand the underlying mechanisms.

The BBB is a critical structure that prevents several substances in the blood from entering the brain. Drugs that can cross the BBB can directly show their effects in the treatment of CNS diseases, including neurodegenerative diseases [20]. Local RAAS blockade in the CNS by BBB-penetrating ACE inhibitors or ARBs, especially in the hippocampus, may promote additional benefits in preventing cognitive impairment and dementia [3]. On the other hand, some drugs may not have protective or beneficial effects on neurons. The present study’s design has allowed investigation of the direct effects of three different types of antihypertensive drugs (all study drugs can cross the BBB) on neuroprotection and ACh release against LPS-induced injury in in vitro settings.

Previous studies illustrated the potential therapeutic effects of ARBs, especially telmisartan, against neurotoxicity [21-23]. In a study, all ARBs showed neuroprotective effects against glutamate-induced neurotoxicity; however, the effect of telmisartan was superior to other ARBs, including candesartan, losartan, and valsartan [21]. The investigators explained this difference through the peroxisome proliferator-activated receptor-gamma (PPARγ) activation provided by telmisartan [21]. The relationship between the PPARγ agonist effect and the reduction in LPS-induced inflammation was also reported in studies in human monocytes [22] and a neuroinflammation model of an LPS-induced IMR-32 cell line [23]. The results of the present study support the neuroprotective effect of telmisartan. Among all the study drugs, telmisartan showed the best neuroprotection (based on cell viability) against LPS-induced injury in differentiated SH-SY5Y cells. Moreover, telmisartan in 5 and 10-µg/mL concentrations provided cell viability values similar to the control.

Perindopril, an ACE inhibitor, is in a different class of RAAS inhibitors and crosses the BBB [24]. In a rat neuron culture study, it was shown that perindopril could be a protective agent against glutamate-induced toxicity [25]. In another preclinical study, perindopril treatment was reported to potentially protect against LPS-induced injury by suppressing neuroinflammation and modulating brain-derived neurotrophic factor (BDNF) and oxido-nitrosative stress [26]. However, the neuroprotective effects of ACE inhibitors may not be at the same level as ARBs. In our study, perindopril in all given concentrations could not provide similar neuroprotection compared with telmisartan. Additionally, the neuroprotective effect of 10 µg/mL telmisartan was superior to 10 µg/mL perindopril (p=0.006), 5 µg/mL perindopril (p=0.001), and 1 µg/mL perindopril (p=0.001). In a very recent clinical study, the effects of candesartan (an ARB) and lisinopril (an ACE inhibitor) on cognitive functions were evaluated. When compared with lisinopril treatment, candesartan treatment was reported to have superior outcomes, including executive functions and memory, and this difference was independent of blood pressure levels [27]. When compared with ACE inhibitors or other cardiovascular drugs, the superior effects of ARBs on the incidence and progression of dementia and AD were also reported in earlier studies [19]. The potential superior effects of ARBs on cognitive functions can be explained by their mechanism of action. ARBs block AT1 receptors (the effects mediated by AT1 receptors of angiotensin II include vasoconstriction, endothelial dysfunction, neurodegeneration, and other potentially harmful effects) and stimulate AT2 receptors (the effects of AT2 receptors counteract the effects of AT1 receptors). Moreover, recent findings support that RAAS mid-products, such as angiotensin IV and angiotensin 1-7, may provide some additional benefits to cognitive functions. Overall, ARBs are the only antihypertensive drugs that provide AT1 receptor blockade and increase all RAAS-related products [4]. In our study, the neuroprotective effect of telmisartan was superior and more significant compared with perindopril. In the present study, telmisartan also provided a concentration-dependent reduction in TNF-α, IL-1β, and NFκB expression against LPS-induced neuroinflammation. This finding suggests that the superior benefit of telmisartan has come along with the suppression of LPS-induced neuroinflammation. Although it was not evaluated in our study, a decrease in the accumulation of amyloid-beta peptide and phosphorylated tau by administration of low-dose telmisartan was also reported in another preclinical study [28].

One of the most remarkable results of the present study was the lack of a neuroprotective effect with nebivolol administration against LPS-induced injury. Moreover, 5 and 10 µg/mL concentrations of nebivolol decreased cell viability, even though it was statistically nonsignificant. Additionally, among all the study drugs, only nebivolol at the 10 µg/mL concentration caused a significant decrease in cell viability in the absence of LPS stimulation. This result can only be explained by the direct effects of nebivolol. The main mechanism of action of nebivolol and other beta-blockers is based on the inhibition of sympathetic activity via beta-receptor blockade; however, the potential cytotoxic effect of nebivolol should not be ignored and this potential effect requires further investigation, especially in long-term treatment.

As a promising beta-blocker in the treatment of coronary artery disease, nebivolol was reported to inhibit the proliferation and moderately induce apoptosis in human coronary smooth muscle cells and endothelial cells [29]. The findings in previous studies may support additional beneficial effects of nebivolol in the treatment of coronary artery disease. However, for neurodegenerative diseases, the non-protective and cytotoxic effects of beta-blockers may be harmful, especially in long-term treatment. As discussed before, the results of some clinical studies have also shown that selective beta-blockers may worsen cognitive functions [6,7]. The potential cytotoxic effects of nebivolol on neuron-like cells in our study and also other beta-blockers, especially those having BBB-crossing properties, require special emphasis in terms of neurodegenerative diseases. Regarding the findings of the present study, the effects of long-term use of nebivolol and other beta-blockers on cognitive functions should be thoroughly investigated in clinical studies.

ACh is an important neurotransmitter and reduced levels of ACh are associated with the development of dementia and cognitive impairment. In earlier preclinical studies, a link between the cholinergic system, RAAS, and neuroinflammation, and the protective effects of RAAS inhibitors against LPS-induced neuroinflammation through angiotensin II AT1 receptor blockade and ACE inhibition have been demonstrated [30]. In the present study, in addition to cell viability, the effects of the study drugs on ACh levels were evaluated to provide new insights into the association between the cholinergic system and the effects of antihypertensive drugs. After LPS-induced injury, in all given concentrations, telmisartan provided a statistically significant increase in ACh levels that was almost the same as the control, whereas the changes with nebivolol and perindopril were nonsignificant. It can be concluded that telmisartan may also provide maintenance of ACh release against LPS-induced injury, alongside neuroprotective effects. This finding could support a hypothesis that suggested a possible increase in ACh release related to ARBs [5].

All the drugs in the present study have a different mechanism of action; therefore, the effects of ACE enzymes and angiotensin receptors should be evaluated to understand the role of those pathways in signaling mechanisms in future research. As a limitation of the present study, the effects of the study drugs on ACh levels in the absence of LPS stimulation were not evaluated. Therefore, future studies that test the direct effects of the study drugs on ACh levels may provide new findings. Additionally, future research focusing on the effects of increased concentrations of the study drugs and prolonged duration can be conducted. Lastly, the present study was an in vitro study; therefore, the results should be evidenced by further in vivo preclinical and clinical studies.

## Conclusions

The present study showed that telmisartan potently and directly ameliorated LPS-induced injury and neuroinflammation and provided maintenance of ACh levels. The neuroprotective effect of perindopril was inferior to that of telmisartan. The only drug that showed no neuroprotective effect was nebivolol. Therefore, the protective effects of telmisartan and other ARBs, as well as the potential long-term effects of nebivolol and other beta-blockers on neurodegenerative diseases including cognitive impairment, dementia, and AD, should be thoroughly investigated in clinical studies.
